# Added Value of a Blinded Outcome Adjudication Committee in an Open-Label Randomized Stroke Trial

**DOI:** 10.1161/STROKEAHA.121.035301

**Published:** 2021-10-05

**Authors:** Nadinda A.M. van der Ende, Bob Roozenbeek, Olvert A. Berkhemer, Peter J. Koudstaal, Jelis Boiten, Ewoud J. van Dijk, Yvo B.W.E.M. Roos, Robert J. van Oostenbrugge, Charles B.L.M. Majoie, Wim van Zwam, Hester F. Lingsma, Aad van der Lugt, Diederik W.J. Dippel

**Affiliations:** Departments of Neurology (N.A.M.v.d.E., B.R., O.A.B., P.J.K., D.W.J.D.), Erasmus MC University Medical Center, Rotterdam, the Netherlands.; Radiology and Nuclear Medicine (N.A.M.v.d.E., B.R., O.A.B., A.v.d.L.), Erasmus MC University Medical Center, Rotterdam, the Netherlands.; Public Health (H.F.L.), Erasmus MC University Medical Center, Rotterdam, the Netherlands.; Departments of Radiology and Nuclear Medicine (O.A.B., C.B.L.M.M.), Amsterdam UMC, University of Amsterdam, the Netherlands.; Neurology (O.A.B., Y.B.W.E.M.R.), Amsterdam UMC, University of Amsterdam, the Netherlands.; Department of Neurology, Haaglanden Medical Center, the Hague, the Netherlands (J.B.).; Department of Neurology, Radboud University Medical Center, Nijmegen, the Netherlands (E.J.v.D.).; Departments of Neurology (R.J.v.O.), Cardiovascular Research Institute Maastricht, Maastricht University Medical Center, the Netherlands.; Radiology and Nuclear Medicine (W.v.Z.), Cardiovascular Research Institute Maastricht, Maastricht University Medical Center, the Netherlands.

**Keywords:** algorithm, clinical trial, ischemic stroke, odds ratio, telephone

## Abstract

Blinded outcome assessment in trials with prospective randomized open blinded end point design is challenging. Unblinding can result in misclassified outcomes and biased treatment effect estimates. An outcome adjudication committee assures blinded outcome assessment, but the added value for trials with prospective randomized open blinded end point design and subjective outcomes is unknown. We aimed to assess the degree of misclassification of modified Rankin Scale (mRS) scores by a central assessor and its impact on treatment effect estimates in a stroke trial with prospective randomized open blinded end point design.

**Methods:**

We used data from the MR CLEAN (Multicenter Randomized Clinical Trial of Endovascular Treatment for Acute Ischemic Stroke in the Netherlands). The primary outcome was the mRS at 90 days. Standardized, algorithm-based telephone interviews to assess the mRS were conducted from a central location by an experienced research nurse, unaware but not formally blinded to treatment allocation (central assessor). Masked reports of these interviews were adjudicated by a blinded outcome committee. Misclassification was defined as an incorrect classification of the mRS by the central assessor. The effect of endovascular treatment on the mRS was assessed with multivariable ordinal logistic regression.

**Results:**

In MR CLEAN, 53/500 (10.6%) of the mRS scores were misclassified. The degree and direction of misclassification did not differ between treatment arms (*P*=0.59). Benefit of endovascular treatment was shown on the mRS when scored by the central assessor (adjusted common odds ratio, 1.60 [95% CI, 1.16–2.21]) and the outcome adjudication committee (adjusted common odds ratio, 1.67 [95% CI, 1.21–2.20]).

**Conclusions:**

Misclassification by the central assessor was small, randomly distributed over treatment arms, and did not affect treatment effect estimates. This study suggests that the added value of a blinded outcome adjudication committee is limited in a stroke trial with prospective randomized open blinded end point design applying standardized, algorithm-based outcome assessment by a central assessor, who is unaware but not formally blinded to treatment allocation.

**Registration:**

URL: https://www.isrctn.com; Unique identifier: ISRCTN10888758.

## Abstract 2

Supplemental Digital Content is available in the text.

Valid outcomes are essential in the evaluation of treatment effect in clinical trials. In stroke trials, the modified Rankin Scale (mRS) is the most commonly used primary outcome measure.^1^ This 7-point ordinal scale describes the degree of global disability or dependence in daily life after stroke (ie, functional outcome).^2^ To obtain reliable mRS scores is challenging in all stroke trials due to the subjective nature causing moderate interobserver agreement.^3^ With the introduction of the prospective randomized open blinded end point (PROBE [prospective randomized open blinded end point]) design,^4^ which is frequently used in stroke trials, reliable outcome assessment became even more challenging. It may be difficult to remain blinded during mRS assessment because patients and their proxies are aware of the treatment they received. This is especially difficult when the treatment contrast between the experimental and control arm is large, for example, in trials that compare an intervention such as endovascular treatment with no intervention. Unblinded assessment of outcomes can lead to systematic (ie, differential) misclassification, which causes biased effect estimates.^5–8^ In addition, incorrect classification of outcomes, also when at random (ie, nondifferential misclassification), can reduce the power of detecting a true treatment effect.^8,9^ Both differential and nondifferential misclassification can result in incorrect conclusions with regard to treatment efficacy.

To reduce differential and nondifferential misclassification, external, blinded outcome adjudication can be used. An outcome adjudication committee consists of a group of independent clinical experts who validate the assessment of outcomes in a randomized controlled trial.^10^ In trials with PROBE design, they also assure blinded outcome assessment by evaluating masked reports. A systematic review and meta-analysis concluded that central adjudication in stroke trials did not have any impact on trial conclusions.^11^ However, site investigators were blinded to treatment allocation in the majority of the included studies, and the studies had predominantly objective outcome measures. Outcome adjudication committees may be most valuable in studies in which both the intervention is not delivered in a blinded manner and the outcomes are subjective.^10,12^ The added value of an outcome adjudication committee in trials with PROBE design and a subjective outcome is unknown. We aimed to assess the degree of misclassification of mRS scores by a central assessor compared with mRS scores of an outcome adjudication committee and its impact on treatment effect estimates in a stroke trial with PROBE design.

## Methods

For this study, the GRRAS (Guidelines for Reporting Reliability and Agreement Studies) guidelines were followed (Table I in the Data Supplement).^13^

### Data

We used data from the MR CLEAN (Multicenter Randomized Clinical Trial of Endovascular Treatment for Acute Ischemic Stroke in the Netherlands).^14^ In short, MR CLEAN was a phase 3, multicenter, clinical trial with PROBE design that evaluated the efficacy and safety of endovascular treatment plus usual care (intervention) compared with usual care alone (control) in ischemic stroke patients with a proximal intracranial arterial occlusion in the anterior circulation. All patients or their legal representatives provided written informed consent before randomization. The central medical ethics committee and the research board of each participating center approved the study protocol.^15^ Anonymized trial data and methods that support our study findings are available from the principal investigator (email: mrclean@erasmusmc.nl) upon reasonable request.

### Assessment of the mRS

One experienced research nurse conducted follow-up interviews at 90 days after randomization by telephone at a central location (central assessor) in all 500 trial patients. If a patient was unavailable or unable to answer the questions, a proxy was interviewed, mostly partner, child, or a health care provider. The standardized, algorithm-based telephone interview included assessment of the mRS, Barthel index, and Euroqol5D.^2,16,17^ The research nurse was mRS certified before mRS assessment was started. The research nurse was unaware of treatment allocation but was not considered formally blinded to treatment allocation because the blinding could have been broken during outcome assessment by the patient or proxy.

### Adjudication of the mRS by an Outcome Committee

Adjudication of the mRS was performed by an outcome committee, which consisted of 5 experienced vascular neurologists who were blinded to treatment allocation. Masked reports of the structured interviews were sent to 2 adjudicators of the outcome committee, who scored the 90-day mRS independently based on the masked reports. The report was extracted from the electronic Case Report Form filled out by the research nurse and included a narrative in words used by the patients, describing their situation in everyday life, which should be included on the blank lines in the Case Report Form (Table II in the Data Supplement). If there was disagreement between the 2 adjudicators of the outcome committee, a third independent adjudicator of the committee gave the final verdict based on all the available information including the mRS score of the central assessor and the other adjudicators of the outcome committee. Misclassification is defined as an incorrect classification of the mRS by the central assessor compared with the final mRS score of the outcome committee. We assumed the score of the outcome committee as reference standard for the correct classification because the outcome committee ensured blinded assessment.

### Statistical Analysis

The trial was analyzed according to the intention-to-treat principle. We compared baseline characteristics of patients in the intervention arm versus the control arm using descriptive statistics. We described the distributions of mRS scores by treatment allocation as scored by the central assessor and as scored by the outcome committee. Differences in the degree and direction of misclassification over treatment arms were compared with a χ^2^ test.

Treatment effect on the mRS was assessed with adjusted ordinal logistic regression for both the mRS scored by the central assessor and the mRS scored by the outcome committee. Results were expressed as adjusted common odds ratios. Treatment effects were also calculated for all possible cut points on the mRS and are expressed as adjusted odds ratios. We adjusted for similar covariates as in the MR CLEAN trial: age; National Institutes of Health Stroke Scale score at baseline; time from stroke onset to randomization; status with respect to previous stroke, atrial fibrillation, and diabetes; and occlusion of the internal carotid artery terminus (yes versus no). Time from onset to randomization was missing for 2 (0.4%) patients and was imputed with single imputation. Statistical analyses were performed with R statistical software (version 3.5.2).

## Results

**Figure 1.**
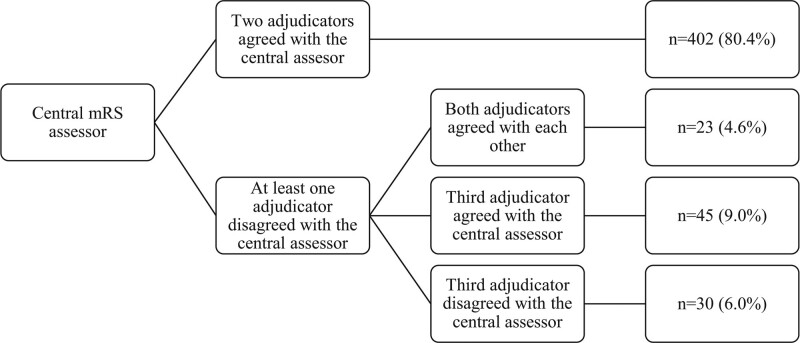
**Agreement between the central assessor and adjudicators of the outcome committee.** mRS indicates modified Rankin Scale.

All 500 patients in MR CLEAN were included in this study. Distribution of baseline characteristics was similar in the intervention and control arm (Table 1). The mRS scores at 90 days as scored by the central assessor and by the outcome committee were available in all patients. In 98/500 (19.6%) patients, at least one of the 2 adjudicators of the outcome committee disagreed with the central assessor (Figure 1). Both adjudicators of the outcome committee disagreed with the central assessor but agreed with each other in 23/500 (4.6%) patients. Hence, a third adjudicator was not required. A third adjudicator was required in 75/500 (15%) patients. When a third adjudicator was required, the final mRS score differed from the mRS score of the central assessor in 30/500 (6.0%) patients. In total, 53/500 (10.6%) of the final mRS scores were misclassified by the central assessor.

**Table 1.**
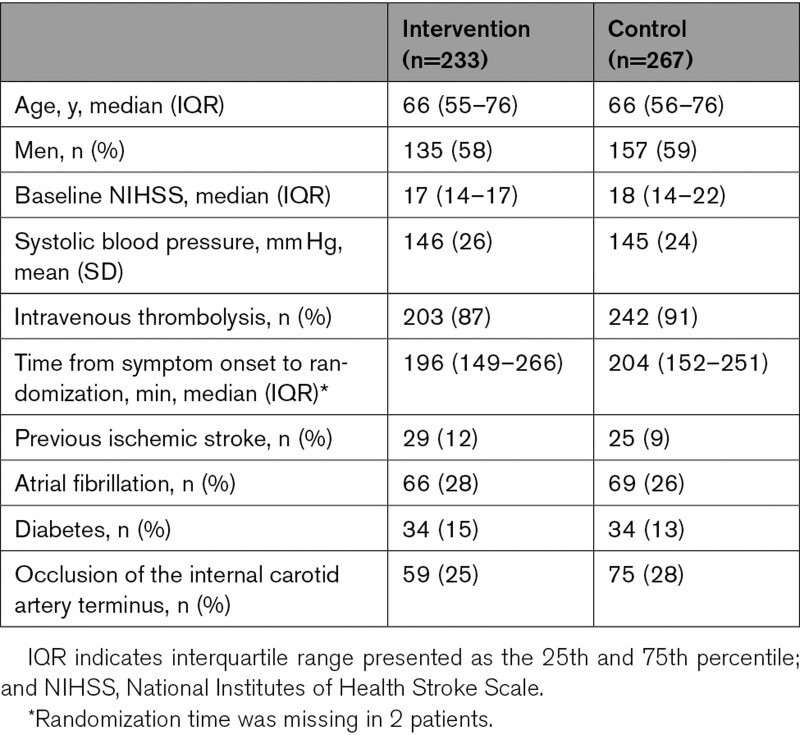
Baseline Characteristics According to Treatment Allocation

### Misclassification of the mRS

**Figure 2.**
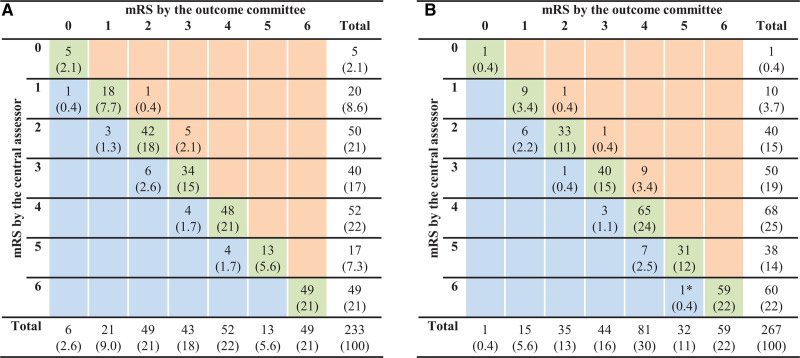
**Cross-tabulation of mRS (modified Rankin Scale) scores by the central assessor and outcome committee according to treatment allocation.** Values are numbers (percentages): data of the intervention arm (**A**) and the control arm (**B**). The green cells indicate no misclassification, the orange cells indicate misclassification towards better mRS scores by the central assessor, and the blue cells indicate misclassification towards worse mRS scores by the central assessor. *This patient died at 90+1 d after treatment. The outcome committee assigned a score of 5 on the mRS to the patient because the patient was alive at exactly 90 d.

Figure 2 shows a cross-tabulation of the mRS scored by the central assessor and the mRS score of the outcome committee according to treatment allocation. The mRS scores were never misclassified by >1 point by the central assessor. The percentage of total agreement (diagonal green cells) was 209/233 (89.7%) in the intervention arm and 238/267 (89.1%) in the control arm. Misclassification by the central assessor leading to higher mRS scores was 7.7% (18/233) in the intervention arm and 6.7% (18/267) in the control arm. Misclassification by the central assessor leading to lower mRS scores was 2.6% (6/233) in the intervention arm and 4.1% (11/267) in the control arm. There was no difference in degree and direction of misclassification between treatment arms (*P*=0.59; Table 2).

**Table 2.**
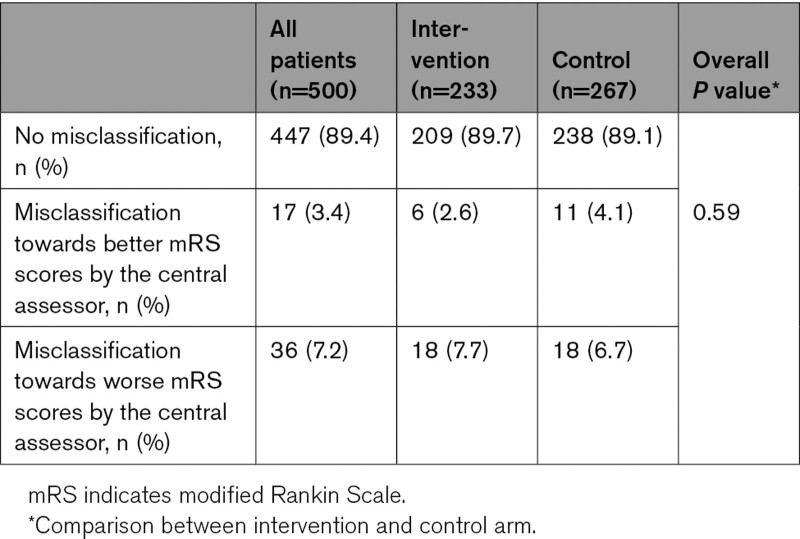
Misclassification of mRS Scores by the Central Assessor

### Impact of mRS Misclassification on Treatment Effect

**Figure 3.**
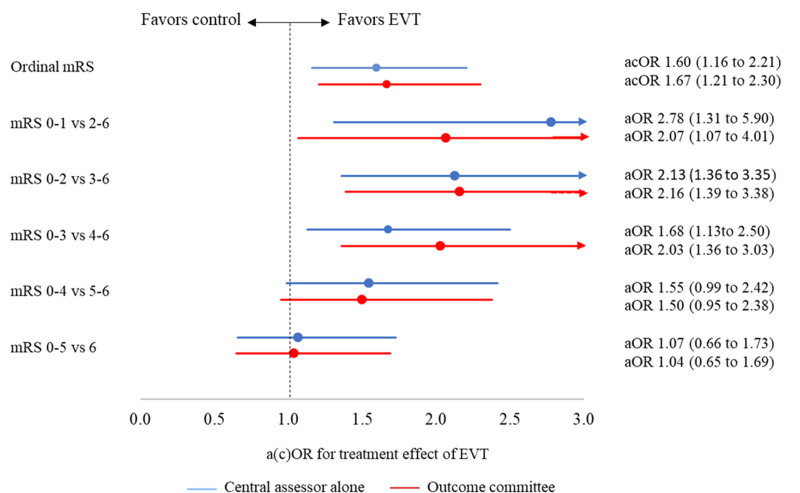
**Treatment effect of endovascular treatment (EVT) on the modified Rankin Scale (mRS) according to the central assessor alone and the outcome committee.** acOR indicates adjusted common odds ratio; aOR, adjusted odds ratio; and cOR, common odds ratio. *Values were adjusted for age; National Institutes of Health Stroke Scale score at baseline; time from stroke onset to randomization; status with respect to previous stroke, atrial fibrillation, and diabetes; and occlusion of the internal carotid artery terminus (yes/no).

Benefit of endovascular treatment on the mRS was shown by both the central assessor (adjusted common odds ratio, 1.60 [95% CI, 1.16–2.21]) and the outcome committee (adjusted common odds ratio, 1.67 [95% CI, 1.21–2.30]; Figure 3). Benefit of endovascular treatment was also shown for excellent outcome (mRS score of 0–1 versus 2–6) and functional independence (mRS score of 0–2 versus 3–6) on both the mRS as scored by the central assessor and the mRS as scored by the outcome committee. Treatment effect estimates on the other cut points pointed towards benefit of endovascular treatment and were similar for both the mRS as scored by the central assessor and the mRS as scored by the outcome committee.

## Discussion

We evaluated misclassification of outcomes by one trained central assessor and its influence on treatment effect estimates of a subjective outcome to assess the added value of a blinded outcome adjudication committee in a stroke trial with PROBE design. Misclassification of the mRS by the central assessor was small, nondifferential and did not influence treatment effect estimates.

We only found evidence of nondifferential misclassification. In our study, compared with the adjudication committee’s assessment, the central assessor more often assigned higher mRS scores than lower mRS scores, 7.2% versus 3.4%, respectively. The impact of nondifferential misclassification patterns has been assessed in simulation studies of patients with traumatic brain injury.^9,18^ These studies showed that nondifferential misclassification is an important problem because it often affects the precision of the effect estimate, which reduces the power to detect the true treatment effect.^8,18–20^ These effects can affect trial conclusions, especially when treatment effect estimates are small. This was not confirmed in our study because the nondifferential misclassification was relatively small compared with, for example, the misclassification in simulation studies of traumatic brain injury trials. In these studies, nondifferential misclassification varied from 10% to 20%.^9,18,21^ The degree of misclassification is an important factor that influences the impact of misclassification on trial results and should be taken into account when designing a clinical trial.

In REVASCAT (Randomized Trial of Revascularization With Solitaire FR Device Presenting Within Eight Hours of Symptom Onset), the degree of misclassification was higher than in our study, 32% versus 11%, respectively.^22^ A possible explanation for this difference in degree of misclassification might be that mRS assessment was conducted on-site in REVASCAT and from a central location in our study. Although on-site mRS assessment in clinical trials may be the easiest choice, it has several disadvantages. Local mRS assessment implies multiple assessors, which introduces interobserver variability even with training and the use of structured interviews.^23,24^ In addition, it is easier to ensure that a small number of central assessors are sufficiently experienced and have received appropriate and consistent training than for—a large number of—site assessors. Furthermore, one can argue that central assessors may be more rigorous to scoring functional outcome of patients than local assessors because mRS scores are also used as outcome indicators to assess the quality of stroke care. Additionally, central adjudicators do not have access to other sources of information related to treatment allocation. The likelihood of unblinding is larger for local investigators than for central adjudicators, which makes the likelihood of misclassified outcomes larger. This is supported by the secondary analysis in REVASCAT, in which a larger treatment effect was observed with local evaluations.^22^ For these reasons, it is important to take the outcome assessment method and number of assessors into account when estimating the misclassification rate.

**Figure 4.**
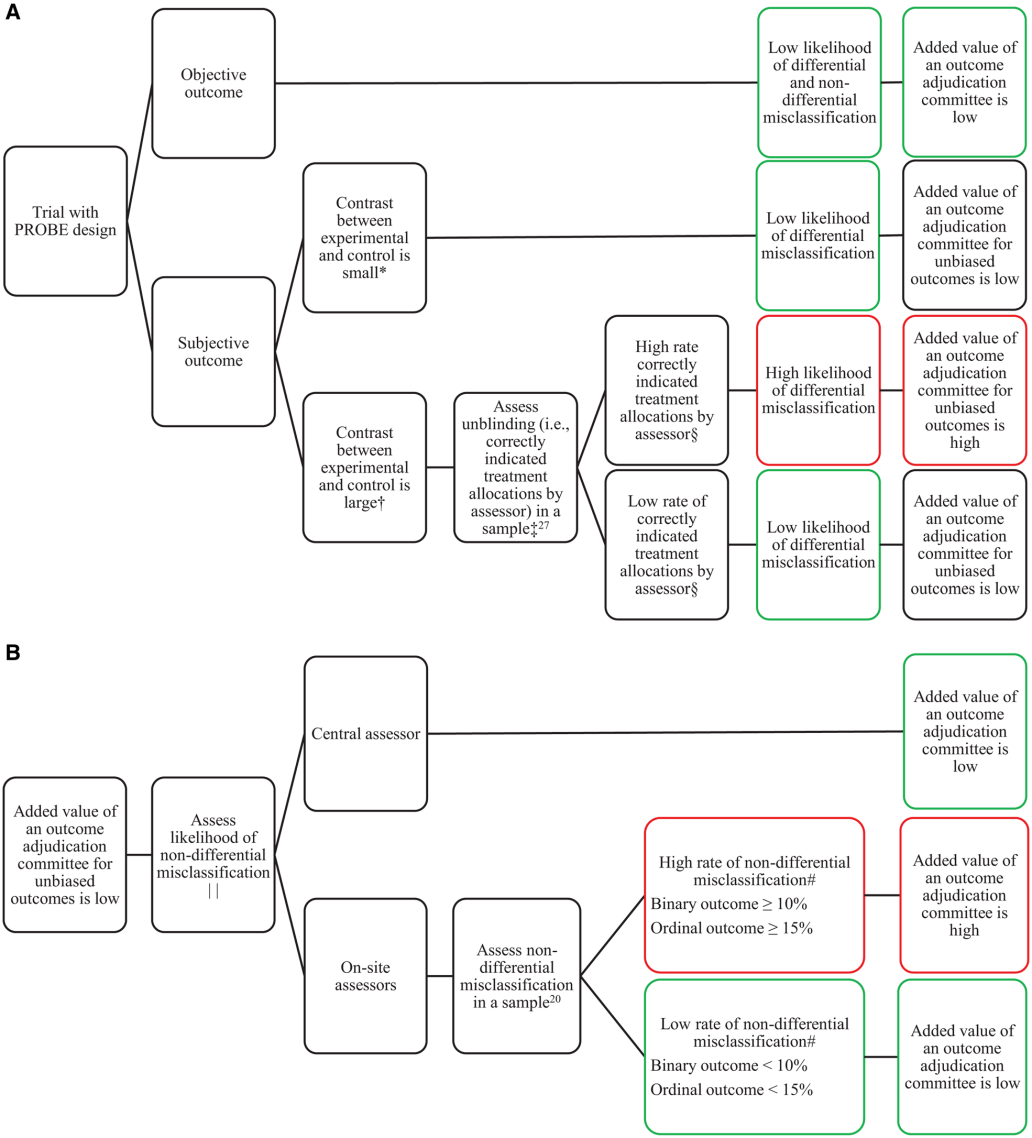
**Flowchart to assess added value of an outcome adjudication committee in trials with prospective randomized open blinded end point (PROBE) design.** Flowchart for differential misclassification (**A**) and nondifferential misclassification (**B**). *The likelihood of unblinding during outcome assessment is low. †The likelihood of unblinding during outcome assessment is high. ‡The likelihood of unblinding is lower for a central assessor than for on-site assessors. §The acceptable rate of correctly indicated treatment allocations by the assessor depend on the number of treatment arms. For example, in a trial with 2 treatment arms, the assessor should not be able to indicate the correct treatment allocation in significantly more than 50% of the cases.^27^ ∥The nondifferential misclassification rate can be reduced by standardized outcome assessment. #The impact of nondifferential misclassification also depends on the size of the treatment effect.

Another factor that influences the effect of misclassification on the estimated treatment effect is the type of outcome measure. Objective outcomes are at a low risk of both differential and nondifferential misclassification and, therefore, the added value of an outcome adjudication committee for objective outcomes is low.^12^ Furthermore, the data type and analysis of the outcome measure is an important factor. For example, there are several differences between binary and ordinal outcomes that influence the degree and effect of misclassification. First, although we did not observe this in our study, ordinal outcomes can be misclassified by more than one level (eg, mRS score of 1 to 3). Second, the likelihood of misclassification can differ over the levels of the ordinal outcome. Because misclassification of deceased patients (ie, mRS score of 6) is unlikely, the proportion of misclassified patients will increase when fewer patients have died. When the proportion of deceased patients differs between the treatment arms, the degree of misclassification between treatment arms will differ. Finally, trials with ordinal outcomes need greater misclassification to alter trial results than trials with binary outcomes.^18,19^ In our study, the degree of misclassification varied according to the mRS scores. This led to unpredictable changes in treatment effect estimates across the different cut points of the mRS. In addition to the many disadvantages of dichotomizing outcome measures,^25^ the observation that treatment effect estimates per cut point of the mRS are affected more and differently by misclassification than the treatment effect estimates on the full ordinal mRS is yet another argument against dichotomizing outcome measures. Figure 4 provides a flowchart to assess the added value of an outcome adjudication committee for differential misclassification and nondifferential misclassification in trials with PROBE design.

Our study has several limitations. First, this study is a post hoc analysis. MR CLEAN was not powered to analyze the effects of misclassification by the central assessor but to detect an effect of endovascular treatment. However, this study was twice as large as the secondary analysis in REVASCAT.^22^ Second, mRS assessment by a central assessor is always telephone-based, which provides less information than in-person assessment, for example, due to lack of visual clues. Nevertheless, telephone assessments have a good agreement with in-person assessments.^26^ More importantly, we do not expect that central telephone-based assessments influenced our results because all mRS assessments were telephone-based, independent of the patients’ condition and ability to visit the hospital. Another limitation is that the outcome committee scored the mRS based on masked reports of the structured mRS assessments by the central assessor, which makes adjudication by the outcome committee dependent of the primary assessment by the central assessor. Therefore, including a narrative of words used by the patients, which describes their situation, is essential for this type of adjudication. In addition, this manner of adjudication is inexpensive, costs little time, and, most importantly, is the most frequently used approach in trials with outcome adjudication. An alternative to overcome this limitation could be that all mRS assessments will be performed by 2 independent assessors, however, this is more expensive, and adequate blinding cannot be assured. Moreover, patients’s answers to the second interview will be influenced by the first interview. Studies that rely on incompletely blinded assessors should routinely test the adequacy of the blind in a rigorous unbiased way as part of their quality control, for example, by asking assessors to indicate the treatment allocation.^27^ Additionally, assessors should indicate whether patients or their proxies had brought up information about the treatment. Another approach could be to record outcome assessment and to verify whether patients or proxies had brought up information about the treatment. These tests for adequacy of blinding were not incorporated in MR CLEAN.

To conclude, misclassification by the central assessor was small, randomly distributed over treatment arms, and did not affect treatment effect estimates. This study suggests that the added value of a blinded outcome adjudication committee is limited in a stroke trial with PROBE design applying standardized, algorithm-based outcome assessment by a central assessor, who is unaware but not formally blinded to treatment allocation.

## Article Information

### Acknowledgments

We thank the MR CLEAN (Multicenter Randomized Clinical Trial of Endovascular Treatment for Acute Ischemic Stroke in the Netherlands) investigators listed in the Appendix.

### Sources of Funding

The MR CLEAN (Multicenter Randomized Clinical Trial of Endovascular Treatment for Acute Ischemic Stroke in the Netherlands) was partly funded by the Dutch Heart Foundation and by unrestricted grants from Angiocare BV, Medtronic/Covidien/EV3, MEDAC gmbh/LAMEPRO, Penumbra Inc, Stryker, and Top Medical/Concentric. All funding sources had no role in the study design and conduct; collection, management, analysis, and interpretation of data; preparation, review, or approval of the article; and decision to submit the article for publication.

### Disclosures

Drs Dippel and van der Lugt report funding from the Dutch Heart Foundation, Brain Foundation Netherlands, The Netherlands Organisation for Health Research and Development, Health Holland Top Sector Life Sciences & Health, and unrestricted grants from Penumbra Inc, Stryker European Operations BV, Medtronic, Thrombolytic Science, LLC, and Cerenovus for research, all paid to institution. Dr van Zwam reports speaker fees from Stryker and Cerenovus, paid to institution. Dr Majoie is a recipient of research grants from CVON/Dutch Heart Foundation, European Commission, Dutch Healt Evaluation Program, TWIN Foundation and Stryker, paid to the institution; and is a minority shareholder of Nico-lab. Dr Roos reports being a minority shareholder of Nico-lab. The other authors report no conflicts.

### Supplemental Materials

Online Tables I–II

## Supplementary Material



## APPENDIX

MR CLEAN Investigators: Olvert A. Berkhemer, MD, Department of Radiology, Amsterdam UMC, Location AMC, University of Amsterdam, the Netherlands and Department of Neurology, Erasmus MC University Medical Center Rotterdam, the Netherlands; Puck S.S. Fransen, MD, Department of Neurology, Erasmus MC University Medical Center Rotterdam, the Netherlands and Department of Radiology, Erasmus MC University Medical Center Rotterdam, the Netherlands; Debbie Beumer, MD, Department of Neurology, Erasmus MC University Medical Center Rotterdam, the Netherlands and Department of Neurology, Maastricht University Medical Center and Cardiovascular Research Institute Maastricht (CARIM), the Netherlands; Lucie A. van den Berg, MD, Department of Neurology, Amsterdam UMC, Location AMC, University of Amsterdam, the Netherlands; Hester F. Lingsma, PhD, Department of Public Health, Erasmus MC University Medical Center Rotterdam, the Netherlands; Albert J. Yoo, MD, Department of Radiology, Massachusetts General Hospital, Boston, United States of America; Wouter J. Schonewille, MD, Department of Neurology, Sint Antonius Hospital, Nieuwegein, the Netherlands; Jan Albert Vos, MD, PhD, Department of Radiology, Sint Antonius Hospital, Nieuwegein, the Netherlands; Paul J. Nederkoorn, MD, PhD, Department of Neurology, Amsterdam UMC, Location AMC, University of Amsterdam, the Netherlands; Marieke J.H. Wermer, MD, PhD, Department of Neurology, Leiden University Medical Center, the Netherlands; Marianne A.A. van Walderveen, MD, PhD, Department of Radiology, Leiden University Medical Center, the Netherlands; Julie Staals, MD, PhD, Department of Neurology, Maastricht University Medical Center and Cardiovascular Research Institute Maastricht (CARIM), the Netherlands; Jeannette Hofmeijer, MD, PhD, Department of Neurology, Rijnstate Hospital, Arnhem, the Netherlands; Jacques A. van Oostayen, MD, PhD, Department of Radiology, Rijnstate Hospital, Arnhem, the Netherlands; Geert J. Lycklama a Nijeholt, MD, PhD, Department of Radiology, MC Haaglanden, the Hague, the Netherlands; Jelis Boiten, MD, PhD, Department of Neurology, MC Haaglanden, the Hague, the Netherlands; Patrick A. Brouwer, MD, Department of Radiology, Erasmus MC University Medical Center Rotterdam, the Netherlands; Bart J. Emmer, MD, PhD, Department of Radiology, Erasmus MC University Medical Center Rotterdam, the Netherlands; Sebastiaan F. de Bruijn, MD, PhD, Department of Neurology, HAGA Hospital, the Hague, the Netherlands; Lukas C. van Dijk, MD, Department of Radiology, HAGA Hospital, the Hague, the Netherlands; L. Jaap Kappelle, MD, PhD, Department of Neurology, University Medical Center Utrecht, the Netherlands; Rob H. Lo, MD, Department of Radiology, University Medical Center Utrecht, the Netherlands; Ewoud J. van Dijk, MD, PhD, Department of Neurology, Radboud University Medical Center, Nijmegen, the Netherlands; Joost de Vries, MD, PhD, Department of Neurosurgery, Radboud University Medical Center, Nijmegen, the Netherlands; Paul L.M. de Kort, MD, PhD, Department of Neurology, Sint Elisabeth Hospital, Tilburg, the Netherlands; Willem Jan J. van Rooij, MD, PhD, Department of Radiology, Sint Elisabeth Hospital, Tilburg, the Netherlands; Jan S.P. van den Berg, MD, PhD, Department of Neurology, Isala Klinieken, Zwolle, the Netherlands; Boudewijn A.A.M. van Hasselt, MD, Department of Radiology, Isala Klinieken, Zwolle, the Netherlands; Leo A.M. Aerden, MD, PhD, Department of Neurology, Reinier de Graaf Gasthuis, Delft, the Netherlands; Rene J. Dallinga, MD, Department of Radiology, Reinier de Graaf Gasthuis, Delft, the Netherlands; Marieke C. Visser, MD, PhD, Department of Neurology, Amsterdam UMC, Location VUmc, University of Amsterdam, the Netherlands; Joseph C.J. Bot, MD, PhD, Department of Radiology, Amsterdam UMC, Location VUmc, University of Amsterdam, the Netherlands; Patrick C. Vroomen, MD, PhD, Department of Neurology, University Medical Center Groningen, the Netherlands; Omid Eshghi, MD, Department of Radiology, University Medical Center Groningen, the Netherlands; Tobien H.C.M.L. Schreuder, MD, Department of Neurology, Atrium Medical Center, Heerlen,the Netherlands; Roel J.J. Heijboer, MD, Department of Radiology, Atrium Medical Center, Heerlen, the Netherlands; Koos Keizer, MD, PhD, Department of Neurology, Catharina Hospital, Eindhoven, the Netherlands; Alexander V. Tielbeek, MD, PhD, Department of Radiology, Catharina Hospital, Eindhoven, the Netherlands; Heleen M. den Hertog, MD, PhD, Department of Neurology, Medical Spectrum Twente, Enschede, the Netherlands; Dick G. Gerrits, MD, Department of Neurology, Medical Spectrum Twente, Enschede, the Netherlands; Renske M. van den Berg-Vos, MD, PhD, Department of Neurology, Sint Lucas Andreas Hospital, Amsterdam, the Netherlands; Giorgos B. Karas, MD, Department of Radiology, Sint Lucas Andreas Hospital, Amsterdam, the Netherlands; Ewout W. Steyerberg, PhD, Department of Public Health, Erasmus MC University Medical Center Rotterdam, the Netherlands; H. Zwenneke Flach, MD, Department of Radiology, Isala Klinieken, Zwolle, the Netherlands; Henk A. Marquering PhD, Department of Radiology, Amsterdam UMC, Location AMC, University of Amsterdam, the Netherlands and Department of Biomedical Engineering and Physics, Academic Medical Center Amsterdam, the Netherlands; Marieke E.S. Sprengers, MD, PhD, Department of Radiology, Amsterdam UMC, Location AMC, University of Amsterdam, the Netherlands; Sjoerd F.M. Jenniskens, MD, PhD, Department of Radiology, Radboud University Medical Center, Nijmegen, the Netherlands; Ludo F.M. Beenen, MD, Department of Radiology, Amsterdam UMC, Location AMC, University of Amsterdam, the Netherlands; Rene van den Berg, MD, PhD, Department of Radiology, Amsterdam UMC, Location AMC, University of Amsterdam, the Netherlands; Peter J. Koudstaal, MD, PhD, Department of Neurology, Erasmus MC University Medical Center Rotterdam, the Netherlands; Wim H. van Zwam, MD, PhD, Department of Radiology, Maastricht University Medical Center, the Netherlands; Yvo B.W.E.M. Roos, MD, PhD, Department of Neurology, Amsterdam UMC, Location AMC, University of Amsterdam, the Netherlands; Aad van der Lugt, MD, PhD, Department of Radiology, Erasmus MC University Medical Center Rotterdam, the Netherlands; Robert J. van Oostenbrugge, MD, PhD, Department of Neurology, Maastricht University Medical Center and Cardiovascular Research Institute Maastricht (CARIM), the Netherlands; Charles B.L.M. Majoie, MD, PhD, Department of Radiology and Nuclear Medicine, Amsterdam UMC, Location AMC, University of Amsterdam, the Netherlands; and Diederik W.J. Dippel, MD, PhD, Department of Neurology, Erasmus MC University Medical Center Rotterdam, the Netherlands.
