# Supplementary material for: Added Value of a Blinded Outcome Adjudication Committee in an Open-Label Randomized Stroke Trial
Source: Stroke. 2021 Oct 5;53(1):61–9. doi: 10.1161/STROKEAHA.121.035301 (PMC8700318; doi:10.1161/STROKEAHA.121.035301)
Supplement: Supplementary file 1 [file str-53-061-s001.pdf]

SUPPLEMENTAL MATERIAL ON:

The added value of a blinded outcome adjudication committee in an open-label randomized stroke trial

Nadinda A.M. van der Ende, MD;[1,2] Bob Roozenbeek, MD, PhD;[1,2] Olvert A. Berkhemer, MD, PhD;[1-4] Peter J. Koudstaal, MD, PhD;[1] Jelis Boiten, MD, PhD;[5] Ewoud J. van Dijk, MD, PhD;[6] Yvo B.W.E.M. Roos, MD, PhD;[4] Robert J. van Oostenbrugge, MD, PhD;[7] Charles B.L.M. Majoie, MD, PhD;[3] Wim van Zwam, MD, PhD;[8] Hester F. Lingsma, PhD;[9] Aad van der Lugt, MD, PhD;[2] Diederik W.J. Dippel, MD, PhD;[1] on behalf of the MR CLEAN Investigators[10]

Departments of Neurology[1], Radiology and Nuclear Medicine[2], Public Health[9], Erasmus MC University Medical Center, Rotterdam, the Netherlands;

Departments of Radiology and Nuclear Medicine[3], and Neurology[4], Amsterdam UMC, University of Amsterdam, Amsterdam, the Netherlands;

Department of Neurology[5], Haaglanden Medical Center, The Hague, The Netherlands;

Department of Neurology[6], Radboud University Medical Center, Nijmegen, The Netherlands;

Departments of Neurology[7], and Radiology and Nuclear Medicine[8], Cardiovascular Research Institute Maastricht, Maastricht University Medical Center, Maastricht, the Netherlands;

The MR CLEAN Investigators[10] are listed in the Appendix.

Supplemental Table I. Guidelines for Reporting Reliability and Agreement Studies (GRRAS).

Section	Item number	Description	Addressed on page number
Title and Abstract	1	Identify in title or abstract that interrater/intrarater reliability or agreement was investigated.	3
Introduction	2	Name and describe the diagnostic or measurement device of interest explicitly.	5-6
	3	Specify the subject population of interest.	5-6
	4	Specify the rater population of interest (if applicable).	5-6
	5	Describe what is already known about reliability and agreement and provide a rationale for the study (if applicable).	5-6
Methods	6	Explain how the sample size was chosen. State the determined number of raters, subjects/objects, and replicate observations.	6-7
	7	Describe the sampling method.	6-7
	8	Describe the measurement/rating process (e.g. time interval between repeated measurements, availability of clinical information, blinding).	6-7
	9	State whether measurements/ratings were conducted independently.	7
	10	Describe the statistical analysis.	8

Results	11	State the actual number of raters and subjects/objects which were included and the number of replicate observations which were conducted.	8-9 + Figure 1
	12	Describe the sample characteristics of raters and subjects (e.g. training, experience).	8 + Table 1
	13	Report estimates of reliability and agreement including measures of statistical uncertainty.	9 + Table 2-3
Discussion	14	Discuss the practical relevance of results.	10-13
Auxiliary material	15	Provide detailed results if possible (e.g. online)	NA

NA indicates not applicable.

Supplemental Table II. Three month follow-up Case Report Form

Date of three month follow-up:	_ _ - _ _ - _ _ _ _ (dd-mm-yyyy)		Who completed assessment:___________________		
Patient deceased:	○ Yes	○ No			
Date of death:	____________________				
Days in a nursing home after discharge:	____________________days		Length: ________		
Days in a rehabilitation center after discharge:	____________________days				
Days in a geriatric rehabilitation unit after discharge:	____________________days		Smoking on admission? __________		
Have you been re-admitted to a hospital after discharge?	____________________days				
Current residence:					
○ Home	○ Nursing home	○ Other:_____________________			
○ Rehabilitation center	○ Hospital				
○ Geriatric rehabilitation unit	○ Friends/family				
Days in a day care facility after discharge	____________________days				
Hours of speech and language therapy after discharge:	____________________hours				
Hours of physiotherapy after discharge:	____________________hours				
Hours of ergo therapy after discharge?	____________________hours				
Do you feel that you have made a complete recovery from your stroke?			○ Yes	○ No	
Does the person have difficulty reading or writing?			○ Yes	○ No	
Does the person have difficulty speaking or finding the right word?			○ Yes	○ No	
Does the person have problems with balance or coordination?			○ Yes	○ No	
Does the person have visual problems?			○ Yes	○ No	
Does the person have numbness (face, arms, legs, hands, feet)?			○ Yes	○ No	
Has the person experienced loss of movement (face, arms, legs, hands, feet)?			○ Yes	○ No	
Does the person have difficulty with swallowing?			○ Yes	○ No	
Any other problems or difficulties?			○ Yes	○ No	
Is assistance needed for activities of daily living?			○ Yes	○ No	
Is assistance essential for preparing a simple meal?<br>*(For example, able to prepare breakfast or a snack)*			○ Yes	○ No	
Is assistance essential for basic household chores?<br>*(For example, finding and putting away clothes, clearing up after a meal. Excludes chores that do not need to be done every day, such as using a vacuum cleaner.)*			○ Yes	○ No	
Is assistance essential for managing finances?			○ Ja	○ Nee	
Is assistance essential for local transportation?<br>*(Patients may drive or use public transport to get around. Ability to use a taxi is sufficient, provided the person can phone for it themselves and instruct the driver.)*			○ Yes	○ No	
Is assistance essential for local shopping?<br>*(Local shopping: at least able to buy a single item.)*			○ Yes	○ No	
Before stroke was the person looking after family at home?			○ Yes	○ No	
**If yes:** Since stroke has there been a change in their ability to look after family at home?			○ Yes	○ No	
Before stroke, was the person working or seeking work (or studying as a student)?<br>*(If the person was not employed or seeking work before stroke, or the person was retired then indicate 'No')*			○ Yes	○ No	
**If yes:** Since stroke has there been a change in the person's ability to work or study?<br>*(Change in ability to work or study includes loss of employment or reduction in level of responsibility; change in education or problems with study).*			○ Yes	○ No	
Before stroke did the person have regular free-time activities?			○ Yes	○ No	
**If yes:** Since stroke has there been a change in their ability to participate in these activities?			○ Yes	○ No	
Do you live alone?			○ Yes	○ No	
Is there someone who visits you regularly and also helps you with something?			○ Yes	○ No	
Do you have professional help? *(housekeeping or home care)*			○ Yes	○ No	
Are there other illnesses or shortcomings that make you less able to take care of yourself or make you less mobile?			○ Yes	○ No	
Can you easily be home alone for a day?			○ Yes	○ No	
Can you easily be home alone for a week?			○ Yes	○ No	

____
____
____
____
____
____

BARTHEL INDEX

Feeding
- Unable
- Needs help cutting, spreading butter, or requires modified diet
- Independent

Mobility (on level surfaces)
- Immobile or < 50 yards
- Wheelchair independent, including corners, > 50 yards
- Walks with help of one person (verbal or physical) > 50 yards
- Independent (but may use aid, e.g., cane) > 50 yards

Transfer (bed to chair and back)
- Unable, no sitting balance
- Major help (one or two people, physical), can sit
- Minor help (verbal or physical)
- Independent

Stairs
- Unable
- Needs help (verbal, physical, carrying aid)
- Independent

Grooming
- Needs help with personal care
- Independent face/hair/teeth/shaving (implements provided)

Dressing
- Dependent
- Needs help but can do about half unaided
- Independent (including buttons, zips, laces, etc)

Toilet use
- Dependent
- Needs some help, but can do something alone
- Independent (on and off, dressing, whiping)

Bowels
- Incontinent (or needs to be given enemas)
- Occasional accident
- Continent

Bathing
- Dependent
- Independent (or in shower)

Bladder
- Incontinent, or catheterised and unable to manage alone
- Occasional accident
- Independent

EQ5D

Mobility
- I have no problems in walking
- I have some problems in walking
- I am confined to bed

Self-Care
- I have no problems with self-care
- I have some problems with washing or dressing myself
- I am unable to wash or dress myself

Usual activities (e.g. work, study, housework, family or leisure activities)
- I have no problems with performing my usual activities
- I have some problems with performing my usual activities
- I am unable to perform my usual activities

Pain / Discomfort
- I have no pain or discomfort
- I have some moderate pain or discomfort
- I have extreme pain or discomfort

Anxiety / Depression
- I am not anxious or depressed
- I am moderately anxious or depressed
- I am extremely anxious or depressed

Assessment by:
- Patient
- Proxy

MODIFIED RANKIN SCALE

○	0	No symptoms	No symptoms
○	1	Symptoms, no disability	Minor symptoms that do not interfere with lifestyle
○	2	Slight disability	Slight disability, symptoms that lead to some restriction in lifestyle, but do not interfere with the patient's capacity to look after himself.
○	3	Moderate disability	Moderate disability, symptoms that significantly restrict lifestyle and prevent totally independent existence
○	4	Moderately severe disability	Moderately severe disability, symptoms that clearly prevent independent existence though not needing constant attention
○	5	Severe disability	Severe disability, totally dependent patient requiring constant attention day and night.
○	6	Dead	Death

Is the modified Rankin Scale influenced by comorbidities? ○ Yes ○ No

Describe comorbidities:

____

____

____

____

____

____

____

____

____

____

____

____

____

Name: _________________ Date: _________________

Signature: _________________
