# Supplementary figures and images for: Added Value of a Blinded Outcome Adjudication Committee in an Open-Label Randomized Stroke Trial
Source: Stroke. 2021 Oct 5;53(1):61–9. doi: 10.1161/STROKEAHA.121.035301 (PMC8700318; doi:10.1161/STROKEAHA.121.035301)

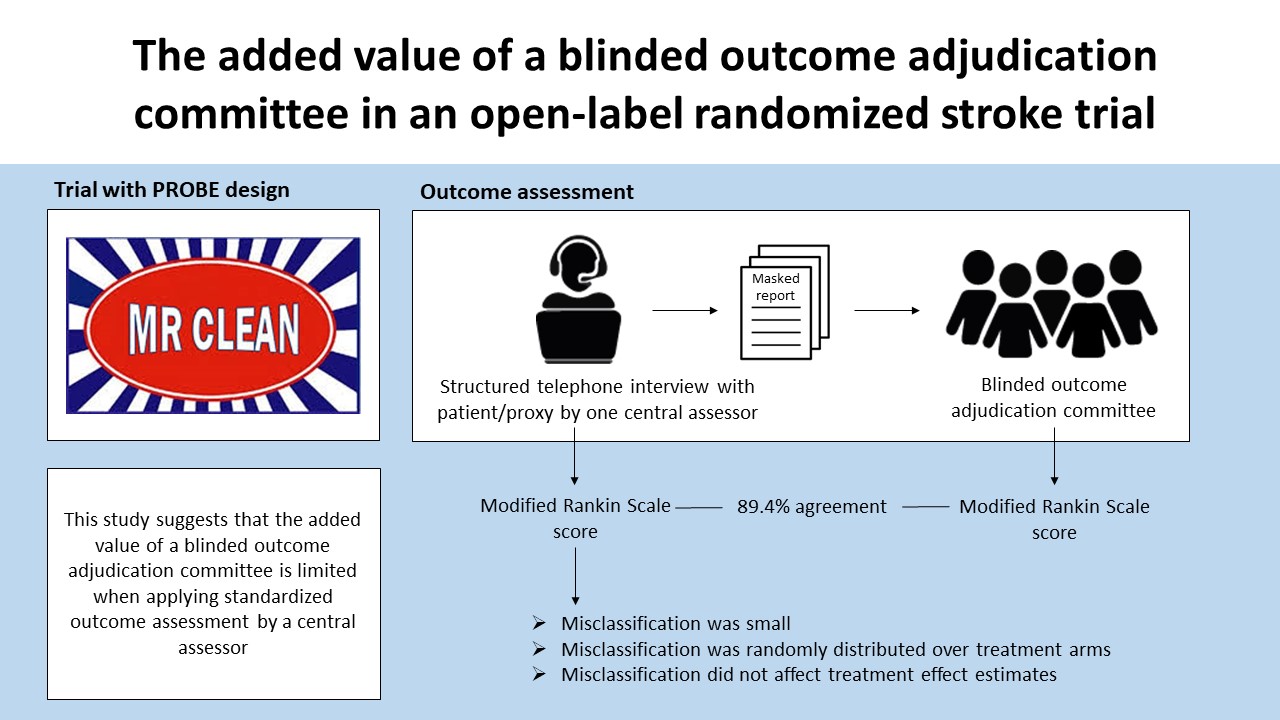

Supplement: Supplementary file 2 [file str-53-061-s002.jpg]
